# Analysis of the Behavior of Fiberglass Composite Panels in Contact with Water Subjected to Repeated Impacts

**DOI:** 10.3390/polym14194051

**Published:** 2022-09-27

**Authors:** Anabelis Carolina Omaña Lozada, José Manuel Arenas Reina, Juan Carlos Suárez-Bermejo

**Affiliations:** 1 ETS de Ingeniería y Diseño Industrial, Universidad Politécnica de Madrid, Rda. de Valencia, 3, 28012 Madrid, Spain; 2 Departamento de Materiales, ETS de Ingenieros Navales, Universidad Politécnica de Madrid, Av. de la Memoria, 4, 28040 Madrid, Spain

**Keywords:** slamming, damage, cyclical impact, fiberglass composite, shear test

## Abstract

One of the most common applications of glass fiber composite materials (GFRP) is the manufacturing of the hulls of high-speed boats. During navigation, the hull of these boats is subjected to repetitive impacts against the free surface of the water (slamming effect), which can cause severe damage to the material. To better understand the behavior of the composite material under this effect, in the present work, an experimental test has been carried out to reproduce the slamming phenomenon in GFRP panels by means of a novel device that allows this cyclic impact to be obtained while the panels are always in contact with water. By means of non-destructive ultrasound inspection in immersion, it has been possible to establish the evolution of the damage according to the number of impacts received by each panel. Destructive tests in the affected zone, specifically shear tests (Iosipescu test), allow determination of the loss of mechanical properties experienced by the material after receiving a high number of impacts in the presence of water (up to 900,000 impact cycles in some panels). The behavior of the material was found to be very different in wet and dry conditions. Under dry conditions, the material loses stiffness as the damage density increases and its shear strength also decreases, as does displacement at maximum load. For wet conditions, the material shows higher displacements at maximum load, while the shear strength decreases with increasing stiffness.

## 1. Introduction

Polymer-based composites have been widely used in many industrial and engineering applications in place of other materials because of their better technical performance. Among their main advantages are their availability and ease of manufacture, high strength, excellent chemical resistance, and low cost. They also have good mechanical behavior (good flexural, tensile, compressive, and impact strength) and high dimensional stability. Therefore, polymer-based composite materials are an excellent alternative to metallic or ceramic materials in automotive, aerospace, marine, construction, etc., applications.

Among this wide range of composite materials, those that incorporate fiber fabrics are of the greatest technological interest. Therefore, the adequate design of fiber-reinforced composite materials allows one to increase the strength and stiffness to exceptionally high values while maintaining a low density. The most widely used in this field are carbon fiber-reinforced polymers (CFRP) and glass fiber-reinforced polymers (GFRP) because they have a high strength/weight ratio, good resistance to corrosion (for chloride ions and chemical environment), high fatigue resistance, no thermal and electrical conductivity, and, in general, a very good performance/cost ratio [1,2].

However, composite materials reinforced with continuous fibers require very careful design and manufacturing to ensure adequate behavior in service and to avoid loss of their properties due to the action of the environment and the loads supported [3,4]. Therefore, in recent years, numerous investigations have been conducted to better understand the behavior of these materials and propose improvements in their design and fabrication. Therefore, Gukendran et al. [5] investigated the effect of the orientation of jute fiber in epoxy matrix composites on the mechanical and thermal properties of the material. Their main conclusion is that a 30° orientation provides the maximum flexural strength. In another line, Ammar et al. [6] have studied the fabrication of composite materials with fiber and plastic as a more environmentally friendly alternative with adequate recycling possibilities. In relation to their mechanical behavior, Ki-Weon Kang et al. [7] have investigated impact damage and strength reduction in a carbon fiber/epoxy and glass fiber/epoxy composite panel structure. Considering temperature, Lopresto et al. [8] evaluated the impact behavior, both at room temperature and at low temperature, of carbon fiber/vinyl resin composite laminates used in the shipbuilding industry. The test reproduced conditions that simulate the impact of a tool falling on the panel mounted on the hull of a ship in contact with water. In this line of impact-resistant material design, Rolfe et al. [9] have analyzed the design of composite panels capable of resisting blast, impact, and high tensile loading. This study was conducted to compare the blast resistance of glass fiber-reinforced polymer (GFRP) with interlayers of polypropylene (PP) and carbon fiber-reinforced polymer (CFRP) with an identical structural core. The results showed that the GFRP panel with PP interlayers prevented the first layer of the panel from cracking and preserved the integrity of the panel. Thus, the design of a composite material must take into account the impacts and structural responses of the panels, the constituent elements, and the behavior and failure mechanics of the fabric and matrix materials used [10].

When composite structures are submerged in water, it is very important to consider the hydrodynamic and hydroelastic effects [11]. These effects can cause the water flow to change due to structural elastic vibrations and the difference in hydrodynamic pressure at particular locations, causing damage to the composite panels. To investigate this situation, several researchers have analyzed the hydroelastic influence, both as a kinematic effect due to the deflection of the composite panel and as a dynamic effect caused by the interaction between water and structure [12,13,14]. Similarly, finite element simulation studies of delamination have been carried out in composite panels that undergo transient elastic deformations of finite plane strain caused by localized water shocks to determine the panel deformations [15,16].

One of the most common applications of fiber-reinforced polymer composites is the manufacturing of the hulls of high-speed boats. The use of these new materials makes it possible to design lighter and stronger boats, which makes it easier to obtain complex geometries and allows one to adapt the mechanical properties of the material to the needs of the design [17]. Ocean structures and ships are subjected to repetitive impacts due to different causes (wave strikes, falling objects, collisions, and ice damage, etc.) which, on several occasions, have resulted in severe damage to marine structures and loss of life and property [18,19].

In this sense, several research works have been carried out in recent years to analyze the behavior of composite materials subjected to these types of impacts. Therefore, Yongyu Duo et al. [20] investigated glass fiber-reinforced polymer (GFRP) and basalt fiber-reinforced polymer (BFRP) rods submerged in different environments (alkaline, acidic, saline solution, etc.) to evaluate their long-term behavior and durability. Additionally, Chenggao Li et al. [21] developed a hybrid fiber-reinforced polymer composite rod (HFRP) that includes a core of carbon fiber-reinforced polymer (CFC) and a glass fiber-reinforced polymer (GFS) lining to be used as retention wires in bridge structures to replace steel cables. To improve the performance of the composite material under water impact, Tomar et al. [22] were inspired by the turtle shell structure to improve the impact resistance of a polymer composite laminate. Specifically, they designed an improved weave composite material to minimize deformation and damage to the composite material when subjected to a low-velocity impact load.

On the other hand, Ammar Elsheik [23] investigated the application of bistable morphological compounds (BMCs) in energy capture to predict and optimize the behavior of these structures using mathematical modeling, neural network-based tool analysis, and metaheuristic optimizers. Studies have also been conducted to determine the permeability of fibrous porous media used in numerous applications. For example, Boqi Xiao et al. [24] presented a fractal model that explicitly relates the Kozeny–Carman constant (KC) to the permeability of fibrous porous media and provides a better understanding of the physical mechanisms that explain fluid transport through these fibrous porous media. Using these ideas, Mingchao et al. [25] designed a fractal model that explicitly relates the microstructural parameters of the porous media and electrokinetic parameters to quantify the effective diffusivity of the electrolyte in the porous media.

The most frequent repetitive impact on marine structures is caused by wave strikes. In particular, when boats move at high speed in the water, they experience a continuous rise and fall process of their bow due to a hydrodynamic effect, which causes the repetitive and continuous impact of the hull against the free surface of the water (a process known as “slamming”) [26,27]. This phenomenon is a critical process in the service behavior of the hull, as it can cause damage at both microscopic and macroscopic levels in the material. Thus, the use of composite materials in the construction of the hull of this type of speedboat requires a design capable of resisting these repeated impacts against the water for periods of time long enough to justify the investment made. The complexity of the phenomenon is due to the fact that when the bottom of the hull penetrates the fluid, at a certain angle between the surface of the hull and the surface of the water, it causes the fluid in the contact region to move at high speed, regardless of the speed of the vessel [28]. In addition, air is trapped between the water and the submerged surface of the vessel, which further aggravates the situation of high pressure peaks experienced in certain regions of the hull. The responses of the materials to this phenomenon, the mechanisms of damage generation in the composite material, and its influence on the structural integrity of vessels have not yet been fully elucidated and remain a topic of interest today [18,19].

The research conducted to seek these answers ranges from conducting tests with local models of certain portions of the ship, to full-ship models that attempt to simulate at full scale the effect of slamming on the vessel. Experiments with complete ship models seek the overall response of the vessel and are the most realistic, but they are very expensive and the test times are very long. Additionally, interpretation of the results of these tests requires computational models to translate the stresses and strains measured at certain points of the structure into pressure peaks in different areas of the hull. With all this, long-term simulations can be performed that attempt to explain the damage caused by the pressure peak on the vessel material and its premature degradation [29]. The hydrodynamic loads that act on the hull of the ship are dynamic in nature and these forces are transferred to the entire structure of the hull [30]. In the case of vessels made of continuous glass fiber and polymeric matrix composite material (GFRP), the slamming phenomenon has the particularity that pressure peaks are converted into energy that is dissipated by the composite material itself, producing different types of damage (cracking of the polymeric matrix, interlayer decohesion, breaking of reinforcement fibers, etc.), which makes it a very complex impact problem to study due to the multitude of physical mechanisms of damage generation and accumulation that come into play.

The material used in the present investigation is the standard material used in fiberglass boats, and its degradation rate throughout its service life is undoubtedly one of the most important parameters in boat design, as it directly affects its cost, load capacity, comfort, and structural integrity, due to the damage produced in the laminate that accumulates in the material at the microstructural level [31,32]. The energy dissipated in the material after impact is not distributed uniformly, but the orthotropy of the material allows the imposed stresses to respond differently in different directions. The stresses and strains of the laminate are not uniform and vary transversely between layers and depend on the type of composite material, the lamination sequence, and the orientation of the reinforcement fibers, making the type of damage produced different for each practical situation and, generally, difficult to predict without a previous experimental basis [15,16,17,18,19,20,21,22,23,24,25,26,27,28,29,30,31,32,33].

However, experimental works on this subject are scarce and normally do not consider the repetitive nature of impacts on the composite material in contact with water. Therefore, in the present investigation, an experimental test has been carried out to reproduce the slamming phenomenon in GFRP panels. For this purpose, a novel device has been designed and manufactured to reproduce this cyclic impact while the panels are always in contact with water. Likewise, the speed at which impacts occur is higher than that which would take place in service conditions at sea, so this device allows one to shorten the duration of the fatigue tests. As is well known, the effect of water on the polymer that constitutes the matrix can result in long-term accelerated degradation of the composite material, causing it to lose stiffness and strength and jeopardizing the structural integrity of the vessel. Therefore, it is essential to perform repeated impact tests in the presence of water and not in dry conditions. By means of non-destructive ultrasound inspection in immersion, it has been possible to establish the evolution of the damage according to the number of impacts received by each panel. Destructive tests in the affected zone, specifically shear tests (Iosipescu test), allow us to analyze the loss of mechanical properties experienced by the material after receiving a large number of impacts in the presence of water.

## 2. Materials and Methods

### 2.1. Materials

**Figure 1:**
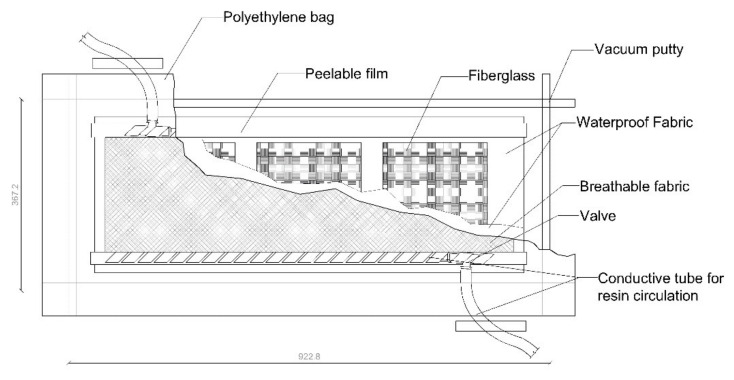
Vacuum bag for the manufacture of GFRP panels by vacuum resin infusion.

For the slamming tests, 220 × 220 mm^2^ panels were prepared with 8 layers of fiber-glass with a lay-up sequence [0°/90°/+45°/−45°]. The first layer is the one that directly receives the impacts through an eccentric cam. The material used was, for reinforcement fibers, 0°/90° twill fabric (UTR 581 T/100, Unique Textiles, Holeslov, Czech Republic, 581 g/m^2^) and +45°/−45° biaxial fabric (X450 E05C, Telateks A.S. Company, Tuzla, Turkey, 450 g/m^2^). With a density of 2550 kg/m^3^, this material has outstanding properties: mechanical (toughness of 1.74 N/tex, tensile strength of 4400 MPa, and elongation at break of 5.2%), thermal (thermal conductivity of 1 W/m·K), dielectric (electric resistivity of 1015 ohm-cm and dielectric dissipation factor of 0.0019 at 105 Hz), and chemical (high resistance to weathering, UV rays, and solvents). A polyester matrix (Crystic U904LVK30, Scott Bader C.L., Wollaston, United Kingdom), specifically designed for infusion, was selected as the composite polymeric matrix. It is a low-viscosity (at 25 °C, viscosity is between 1 and 1.4 dPas), preaccelerated, non-thixotropic, unsaturated orthophthalic polyester resin. Once cured, the resin has a density of 1.1 g/cm^3^ and shows good mechanical properties (tensile strength of 52 MPa and elongation at break of 2%) and thermal properties (its heat deformation temperature is 80 °C). The average thickness of the cured panels is 3 mm. For the manufacture of the panels, the different layers were laminated on a teflon mold (Rongde Mould C.L., Zhuozhou, China), with the reinforcement fibers without polymeric matrix, and vacuum-sealed with a polyethylene bag and vacuum sealant putty around the perimeter. On top of the sheet, a breathing fabric was included, a peelable film was added on both sides of the laminate, and a waterproof fabric to prevent the resin flow from clogging the valve placed to perform the vacuum inside the vacuum bag. The vacuum is maintained by a high-capacity pump that allows the vacuum hose to enter the oven, where gases or volatiles from the curing process can be continuously extracted, controlling the porosity during the curing cycle (Figure 1). Once the necessary vacuum was achieved, the polyester resin was infused until the reinforcing fiber layers were completely soaked.

The panels were cured at room temperature for 24 h. They were then demolded and post-cured in an oven (Dycometal Equipos Control de Calidad S.L., Viladecans, Spain) at 60 °C for 60 min. At the end of the curing time, the oven was turned off and the panels were allowed to cool to room temperature. Each panel was then inspected by ultrasound NDT, using the immersion pulse/echo technique and C-Scan representation, to verify that it was free from manufacturing defects and that the porosity levels were acceptable for panels manufactured using the vacuum resin infusion technique.

### 2.2. Slamming Testing Rig

**Figure 2:**
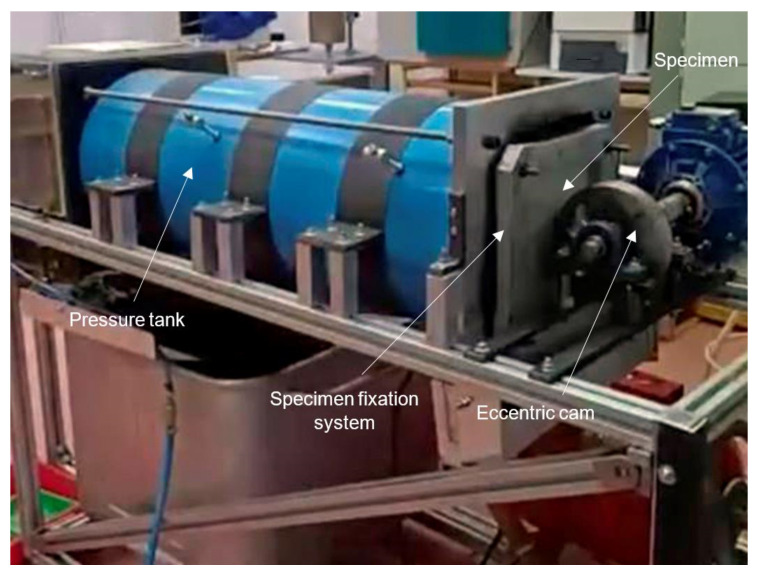
Test rig for the reproduction of slamming cycles, by means of an eccentric cam and pressurized water flow, on the composite test specimen.

The equipment designed to reproduce the slamming cycles in the panels (Figure 2) consists of a water reservoir with fluid pressure control, test specimen clamping frame, and an eccentric cam connected to an electric motor, which regularly impact the panel while rotating at a certain range of speeds (1–4 Hz). The hydraulic system of the machine consists of the following main elements: collector tank, water pump, pressure tank, pressure gauge, pressure line, and discharging chamber. The water required for the test is stored in the collector tank. The pump takes the water from the tank through a pipe and conducts it to the pressure tank, where a three-way valve drives the water through a duct at a flow rate and preselected pressure, to the panel located at the opposite end of the pressure line.

The specimen clamping frame is located on the outer face of the support plate, at one end of the pressure tank, and consists of a flat rubber ring joint. A metal plate solidly fixes the perimeter of the panel to be tested, sealed with a rubber ring, so that when the eccentric cam hits the panel, it does not open a gap between the panel and the plate, causing water leakage, as illustrated in Figure 2. The eccentric cam is made up of an electric motor and a speed variator, which is connected by means of a shaft supported on two bearings. The steel cam, which is keyed to the shaft, has open holes for lightening, so that its mass is appropriately balanced with the center of the shaft and avoids inertial loads due to its eccentric shape. The variator installed allows the motor to work at rotation speeds of 200 to 320 cycles per minute, values at which the assembly is more efficient and the vibrations produced by the equipment when rotating do not affect the assembly of the parts. Rubber gaskets were placed on the bases of the bearings supporting the shaft to dissipate the reaction energy of the cam against the panel and not affect this lateral load to the motor drive assembly. The cam design allows the deformation and relaxation of the panel to gradually press on the contact face and control the duration of the impacts, to accommodate the duration of the pressure peaks characteristic of slamming cycling.

### 2.3. Ultrasonic Assessment

Ultrasonic inspection of each of the manufactured panels is necessary to evaluate the pore content of the original laminate and, fundamentally, to follow the evolution of damage as a function of the number of impact cycles received by the samples. For this purpose, an immersion tank (Tecnitest, Tecnitest Ingenieros, Madrid, Spain) was used, featuring a motorized head and a probe working at a frequency of 5 MHz, with a scanning accuracy of 0.1 to 0.2 mm in both axes, at a maximum inspection speed of 100 mm/s, connected to the integrated data acquisition and management system Masterscan 335 (SDMS, Sonatest, Milton Keynes, United Kingdom). The equipment is both an emitter and receiver; the waves sweep the entire surface of the material looking for discontinuities, such as porosity, microcracks, delaminations, or dry areas, unfilled by the polymeric matrix. The attenuation of the ultrasonic wave can be directly correlated with the level of porosity in the section of material crossed by the ultrasonic beam. The different attenuation levels are represented on an arbitrary color scale (C-Scan) to determine the location of the damage in the panels, before, during, and at the end of the fatigue procedure performed in the slamming simulation equipment. Any area with an attenuation level greater than 18 dB is considered damaged material. A count of bits in this damage condition, performed using open-source image analysis software (Image J, 1.52v, National Institutes of Health, Bethesda, MD, USA), allows quantification of the progression of the damaged area as the number of cycles applied increases.

### 2.4. Impact Tests

**Figure 3:**
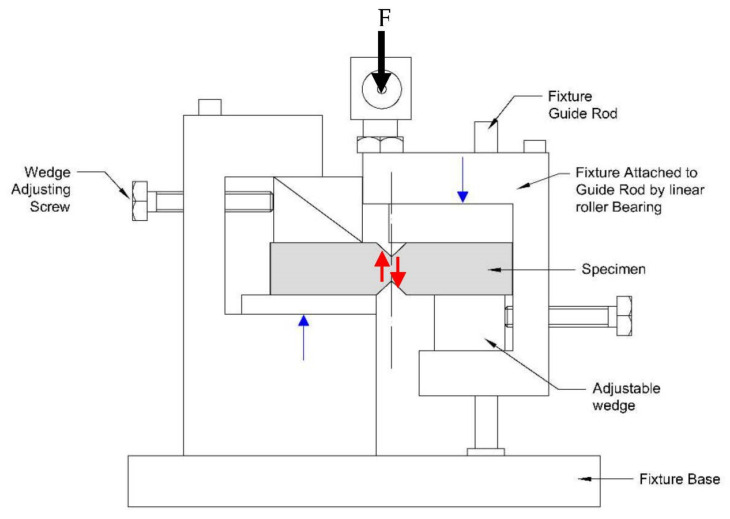
Schematic of the Iosipescu test and shear load applied in the sampled area. (red arrows show the shear load on the specimen and blue arrows the driving direction of the testing machine).

**Figure 4:**
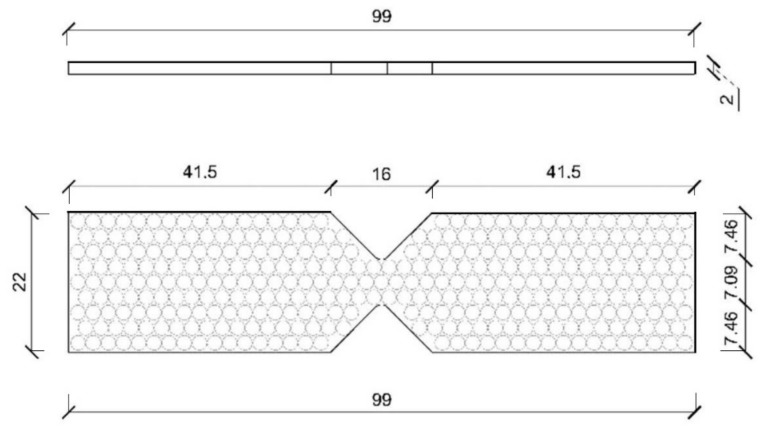
Drawing of the shear specimens extracted from the panels.

To determine the residual strength of the damaged panels and establish a quantitative criterion for the loss of mechanical properties due to slamming, the samples were cut for shear tests using the experimental device known as the Iosipescu test [34]. This test is a convenient procedure to obtain the stiffness and shear strength values of the composite laminate. For this purpose, the test locally imposes, in the area of accumulation of impact damage to be evaluated, a uniform distribution of tangential stresses by means of the applied shear load, so that if two strain gages are mounted at 45° to the direction of the load and perpendicular to each other, we can obtain the values of the shear stress. Figure 3 shows a drawing of the experimental setup, and Figure 4 a drawing of the specimen used in the Iosipescu tests. The area sampled by this type of specimen is small, so property values can be obtained on specimens obtained from different parts of the panel. A total of nine specimens were marked and cut from each panel. Since impacts are applied in the center of the panel and the panel is deformed in bending, with embedment conditions around the perimeter, a simple finite element model is used to determine the stress state in each test specimen, which is a function of the radial distance from the sampled area to the center point of the test panels [35].

## 3. Results

### 3.1. Original, Non-Impacted Panels

**Figure 5:**
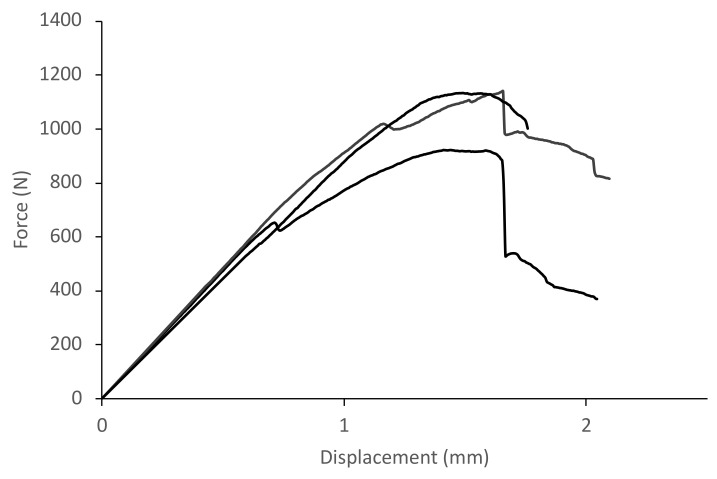
Force–displacement curve for Iosipescu tests of 3 specimens extracted from a non-impacted panel.

The results obtained for the non-impacted panels, i.e., in their original state after being manufactured, are presented in this section. Force–displacement curves were obtained for 10 identical samples, using the Iosipescu test fixture, which had previously been evaluated by ultrasound to verify that they were free of manufacturing defects, to characterize the behavior of the intact panels in shear. The results of three different specimens obtained from the same unimpacted panel are shown in Figure 5. The maximum shear load for these samples is observed to be 1140 N, with a displacement at peak load of 1.72 mm. The straight line before the material begins to show the first signs of failure has a slope of 840 N/mm.

### 3.2. Impact Panels on the Slamming Test Rig

The impact test panels were placed on the slamming test equipment. Pressure was introduced up to 1 atmosphere, but care was taken that the water level in the panel was approximately half its height. In this way, the lower part of the panel (including the central impact area) was tested in a wet condition, while the upper part of the panel remained above the water level and thus in a dry condition. In this way, the entire panel was deformed in flexure by the impacts, but then test samples were obtained from the immersed part and the dry part, to check if contact with water during the slamming impacts implied a difference in behavior in the evolution of the damage. A total of 900,000 impact cycles were applied. A simple visual analysis showed that after 200 impacts, some microcracks were already observed on the surface (Barely Visible Impact Damage, BVID), which became more evident at around 1000 cycles. The first damages, observed as slight white shadows, were located in the areas where they touched the sides of the cam, and then continued to align towards the center of the contact surface. Due to the torque limitation of the cam motor, the cam could only be tested in a rotational speed range between 100 and 126 cycles/min, which means an impact frequency of around 2 Hz.

**Figure 6:**
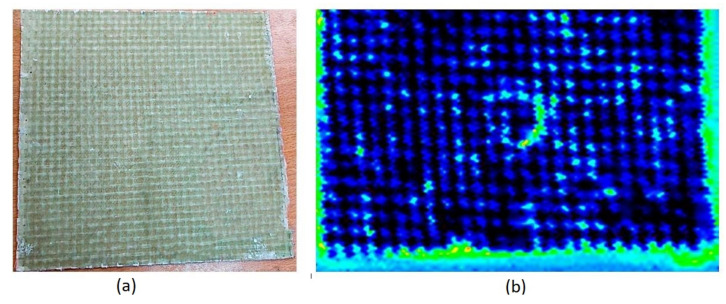
GFRP panel (**a**) and C-Scan representation of the ultrasonic inspection (**b**), showing damage caused by repeated slamming impacts.

The results of the ultrasonic inspection are shown in Figure 6, where it can be seen that the level of porosity resulting from the manufacturing process is minimal (less than 5% of image bits above the damage threshold) and the direction of the reinforcement fibers in the 0/90 fabric layer can be clearly seen. In the central area, where the panel has received the impact of the cam, the generation of damage is observed, which, in subsequent inspections, can be tracked to be propagating. Monitoring of damage with ultrasound analysis made it possible to quantify the increase in microcracks, in order to evaluate the level of damage to the panels after cyclic impacts. With ultrasonic NDT, it is clear that the slamming does not produce a direct delamination phenomenon because the level of pressure is equivalent to a low-energy impact, generating microcracks in the matrix that are directly related to the stiffness residual strength of the composite and therefore to the in-service life span. Evaluation of damage progression at the end of impact cycles indicated levels that did not exceed 60% of bits above the damage threshold. Section 4 presents a more detailed discussion on the damage formation mechanisms in the panels, both for dry and wet conditions.

Nine specimens located in different zones of each panel were marked and cut to perform the Iosipescu shear tests. The specimens obtained from the submerged (wet) half of the panel correspond to odd-numbered codes (S1, S3, S5, and S7), in addition to code S0, which is the one that identifies the central zone where the cam cyclically impacts, and which was also constantly submerged. The specimens with even-numbered identification codes (S2, S4, S6, and S8) correspond to the non-submerged (dry) half of the panel. Each sample was obtained at a certain distance from the center of the panel, so there was a variation in the stress levels produced by the bending of the panel after each impact. The intact (raw) panel is included in all the plots obtained in order to appreciate the variation in mechanical properties due to the damage introduced during slamming fatigue.

**Figure 7:**
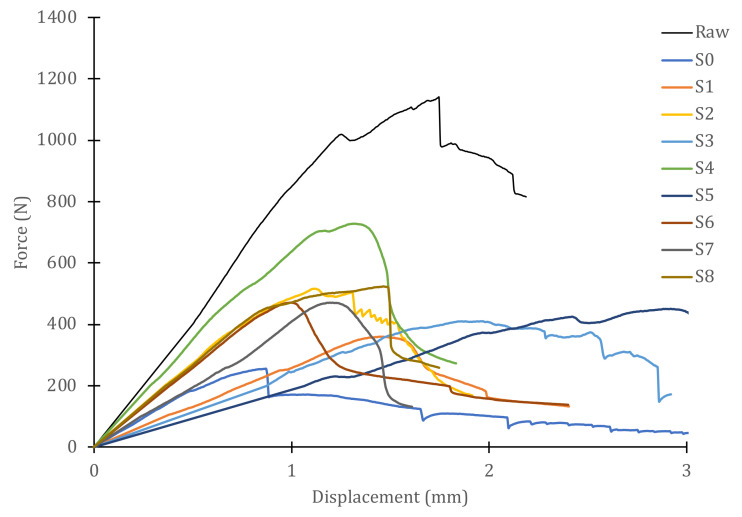
Iosipescu shear test curves for samples (dry/wet) from a GFRP panel with 900,000 cyclic impacts.

**Figure 8:**
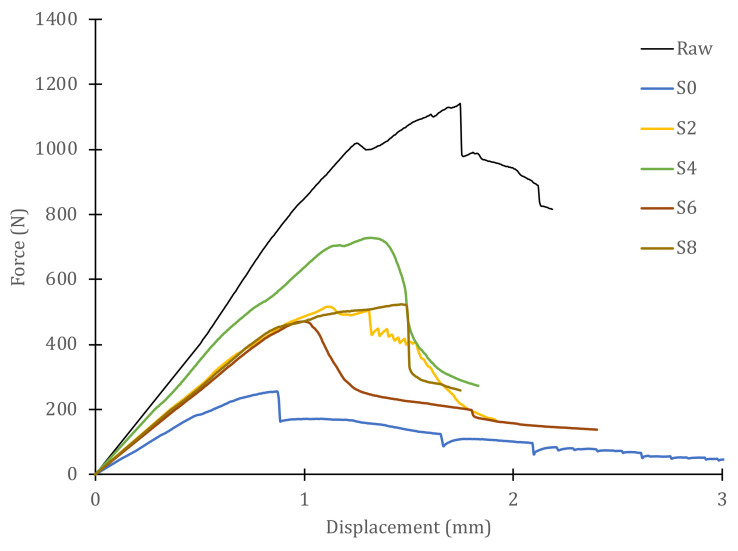
Iosipescu shear test curves for dry samples after 900,000 cyclic impacts.

**Figure 9:**
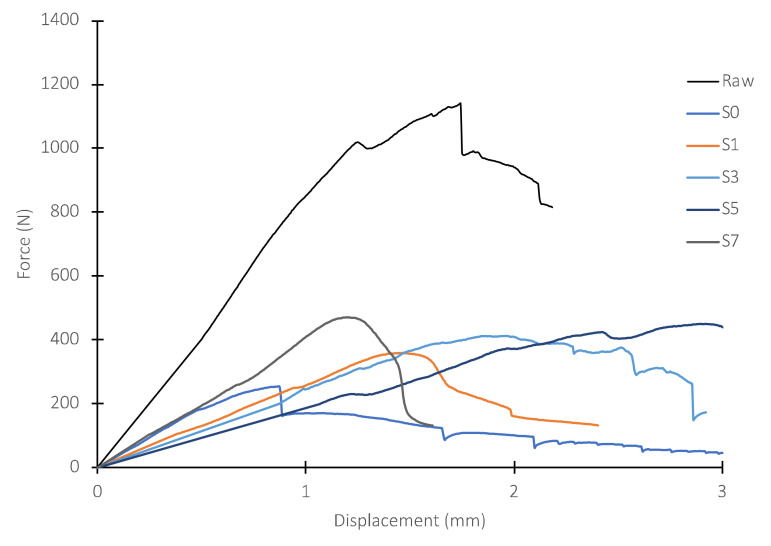
Iosipescu shear test curves for wet samples after 900,000 cyclic impacts.

Figure 7 shows the load–displacement curves for all samples obtained from one of the panels, including the curve corresponding to the intact (raw) material. To better appreciate the behavior of the GFRP panels in dry/wet conditions, Figure 8 shows the samples extracted from the dry area of the panel. On the other hand, Figure 9 includes the plots corresponding to the samples obtained from the area of the panel that was immersed during the slamming impact cycles. For both cases, the curve of the material in its original state, not subjected to slamming impacts, and the curve obtained from the central zone, where the direct impact of the cam is received (S0), are included for reference.

In the case of specimens of dry material, the curves are half-way between those for intact material (highest peak load) and those of the direct impact zone (lowest peak load). The maximum load of each sample and the displacements at which the maximum load occurs vary for every sample. The slopes of the initial straight segments, i.e., the stiffness with which the material behaves in the linear regime, are also different for every sample. As the selected specimens move away from the impact zone, they exhibit higher peak loads, larger displacements at maximum load, with a larger linear zone, and higher stiffness. The individual values are listed in Table 1. However, for specimens obtained from immersed material, the curves are below the curve for the direct impact zone (S0), with stiffnesses equal to or less than those of S0, although the peak loads are higher than those of S0 and, particularly, the displacements at maximum load greatly exceed those of S0. The numerical values of these specimens are also shown in Table 1.

## 4. Discussion

**Figure 10:**
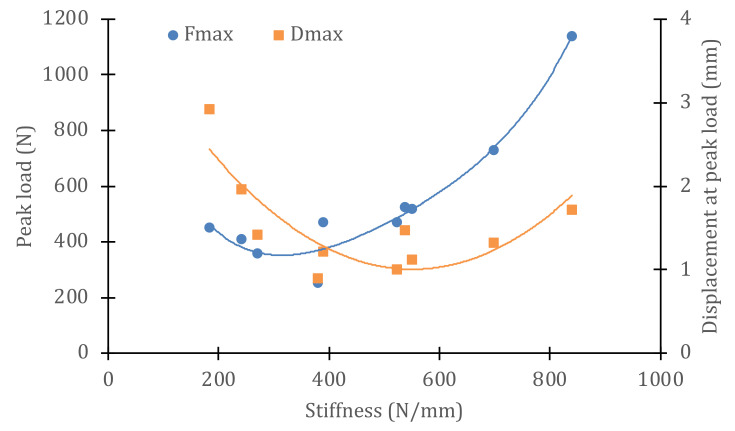
Variation in strength and displacement at maximum load as a function of specimen stiffness, after 900,000 impact cycles, under dry/wet conditions.

The behavior of the material in wet or dry conditions is very different, in view of the results of the Iosipescu shear tests shown in Figure 8, Figure 9 and Figure 10. To better appreciate this behavior, Figure 10 shows a graph with the variations in the maximum loads and the displacements at peak load for each sample. It can be observed that the absolute minimum of both properties corresponds to sample S0—that is, in the immersed zone where the direct impacts of the cam were received. The stiffness value for this sample is 380 N/mm, as can be seen in Table 1. However, the rest of the immersed specimens (S1–S7) have stiffness values similar to or lower than those of S0. Moreover, as the stiffness of the material decreases, the maximum supported load increases and so does the displacement at peak load, always with respect to the reference value of specimen S0. For stiffnesses greater than S0, i.e., specimens taken from dry material but having received the same number of cyclic impacts, the peak load values increase with stiffness (greater distances to the center point of impact) and so do the displacement at peak load values. Obviously, the absolute maximum of these properties corresponds to the behavior of the intact, non-impacted material. The ratio between the maximum (raw) and minimum (S0) values of the stiffness is 45%. However, the residual strength after 900,000 impact cycles (ratio of maximum loads between both extreme values) is only 22% and that of the peak load displacements is 52%. These results show that the slamming process experienced by the material reduces its strength, but not its deformation capacity before failure. Thus, the stiffness is reduced, but not to the same extent as the strength. This information is valuable to be able to estimate the degradation of the vessel material throughout its service life and to decide on the basis of objective criteria when hull repairs should be carried out.

**Figure 11:**
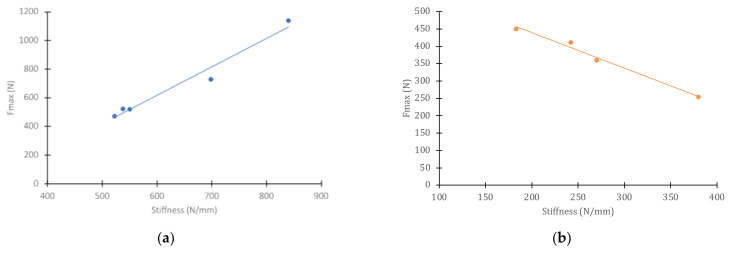
Linear correlation between strength and stiffness after cyclic impact: (**a**) dry material; (**b**) wet material.

In the case of dry specimens, the trend is the reverse, i.e., for specimens farther away from the direct impact zone, which show linear sections with greater slope (higher stiffness), the strength and displacement at maximum load increase. This correlation, practically linear, of strength with the stiffness of the material, for wet and dry specimens, can be seen in Figure 11.

**Figure 12:**
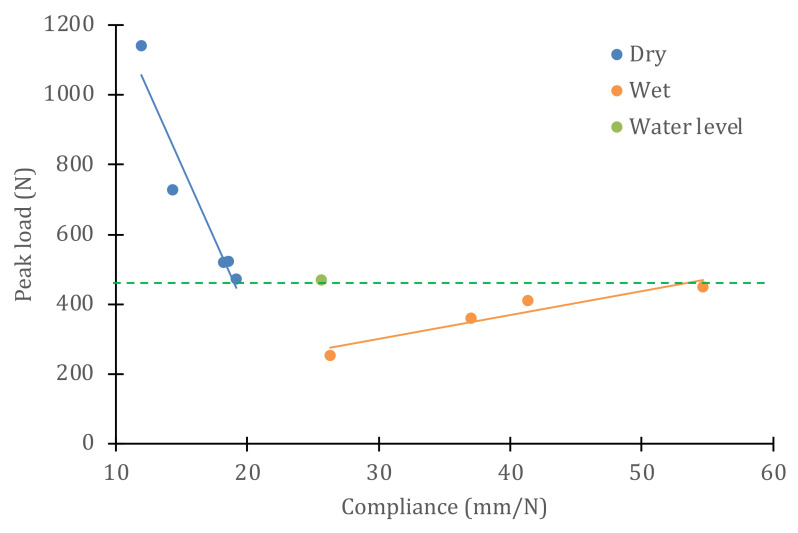
Linear correlation between strength and compliance after cyclic impact, for dry/wet conditions of the samples.

The same information can be represented as the variation in resistance as a function of compliance (inverse of stiffness), which helps to understand the effect of water on the evolution of properties as damage is generated in the material. Figure 12 presents, in the same graph, the results for both the dry/wet conditions of the material, where the horizontal line separates the samples that were submerged from those that were above the water level. For dry material, the maximum load decreases linearly with increasing compliance (slope of −84.3 N^2^/mm), while, for wet material, the maximum load increases linearly with increasing compliance (slope of 6.6 N^2^/mm).

The role of water is a determining factor in the mechanisms of progression of damage in composite materials. Ultrasonic tests showed that the non-impacted panels did not present appreciable porosity, microcracks, or delaminations. However, upon impact, microcracks are generated in the central area of the panel, which grow as the number of impacts increases. This occurs in both submerged and dry material, and the distance to the impact zone is important to determine the level of damage produced. Ultrasound can detect the presence of translaminar and intralaminar microcracks and breaks, which form a damaged area that gradually grows.

Through these microcracks, water enters the GFRP panel, which arrives deeper as the cracks propagate. In the submerged zone, the water is in direct contact and penetrates easily through the network of microcracks, while in the zone above the water level, the water ingress by diffusion is more limited. Water has two well-known effects on the polymer matrix. First, it acts as a plasticizer on the polymer, breaking its chains by hydrolysis and increasing its deformation capacity. On the other hand, the water migrates to the fiber–matrix interface and produces its debonding, by breaking the silane bonds that form the coating of each fiber, to improve its wettability and adhesion to the polymeric matrix. Both effects combined, plasticization of the matrix and separation at the fiber–matrix interface, produce the loss of stiffness observed in the tests.

Nevertheless, the higher compliance of the specimens that remained submerged during the 900,000 impact cycles allows the displacements at maximum load to be much higher than in the intact material. However, the strength of the material is lower in all cases. The best of the submerged specimens (those farthest away from the direct impact zone) fail to reach the worst strength values of the dry specimens (those closest to the direct impact zone), as can be seen in Figure 11. However, in wet material, the polymer matrix is more deformable and is also partially detached from the reinforcing fibers, so it is now the deformation and not the stress that controls the failure initiation process. This justifies the different behavior observed between dry and wet specimens.

In any case, for a proper approach to the fatigue testing of composite panels by repeated impacts, it is important to ensure (when water is an element, in contact with the panels) that the tests are performed with the material in immersion. It is not possible to draw valid conclusions about the durability of these materials when tests are performed (as is often the case) under dry conditions. The mechanisms of the progression of damage are radically different in the presence of water. Tests become more complicated when panels are tested in wet conditions, subjected to a certain hydrostatic pressure, and cyclically impacted over long periods of time. This procedure requires the use of equipment specially designed and built for this purpose, with the consequent complications of keeping the circuit watertight and preventing leaks after each impact, as well as controlling the internal pressure of the pressure chamber, and the frequency and energy of the impacts. However, these complications are necessary if realistic limits are to be placed on the service life of these materials in marine applications.

## 5. Conclusions

The methodology proposed in this research makes it possible to reproduce, under laboratory conditions and using small panels, the slamming impact cycles to evaluate the damage generated in the hulls of fast boats made of GFRP during navigation. Tests are carried out under variable hydrostatic pressure and under cyclic impact conditions that are adjusted in frequency and energy to faithfully reproduce the hull bottom slamming impacts against the free surface of the water. For this purpose, it has been necessary to design and build specific equipment to perform slamming tests on GFRP panels, under conditions representative of the service conditions. The tests have been extended to up to 900,000 impact cycles on some panels.

The damage to the material was assessed by nondestructive ultrasonic inspection. It has been verified, on the one hand, that the starting panels were free of manufacturing defects (beyond those expected for the manufacturing technique used) and, on the other hand, that the damage has progressed after a certain number of impacts. To evaluate the loss of mechanical properties caused in the material by the presence of the accumulated damage, several samples of the panels have been obtained once subjected to the preset number of impact cycles, to be subjected to shear tests in an Iosipescu fixture. In this way, values of shear strength, displacement at maximum load, and stiffness have been obtained in the linear elastic regime, both for specimens in immersion and for others tested in dry conditions.

The main conclusions of the experimental work carried out were the following:

- The behavior of the material was found to be very different in wet and dry conditions. In dry conditions, the material loses stiffness as the damage density increases, and its shear strength also decreases, as does the displacement at maximum load.
- For wet conditions, the material shows higher displacements at maximum load, while the shear strength decreases with increasing stiffness.
- The ratio between the maximum (non-impacted, dry) and minimum (wet, direct impact zone) values of the stiffness is 45%. However, the residual strength after 900,000 impact cycles is only 22% and that of the peak load displacements is 52%.
- The slamming process experienced by the material reduces its strength, but not its deformation capacity before failure. Thus, the stiffness is reduced, but not to the same extent as the strength.

The micromechanisms that explain the difference in behavior between dry and wet material are related to the entry of water through the network of microcracks that form due to cyclic impacts. Water causes hydrolysis of the polymeric chains, plasticizing the matrix of GFRP panels and increasing their deformation capacity, while increasing their compliance. It also contributes to this effect, which is that the water migrates to the fiber–matrix interface and causes it to debond, which also reduces the stiffness of the material. In any case, the performance of slamming tests in the presence of water and hydrostatic pressure is essential to be able to adequately evaluate the degradation of the material throughout its in-service life. The results derived from tests with panels in dry conditions cannot be extrapolated, as they underestimate the degradation rate of the material.

The conclusions obtained in the present work confirm the validity of the proposed methodology and will allow its application in further research on the following aspects:

- Redesign of GFRP panels (different number of layers, different orientation of the glass fiber, different type of resin, etc.) to improve their technical performance against the slamming effect.
- Design of GFRP panels with bioinspired protection layers (based on marine structures existing in nature) and evaluation of their technical performance under slamming impact effects.

## Figures and Tables

**Table 1:** Maximum load, displacement at maximum load, and stiffness values for shear samples, in dry and wet conditions, after 900,000 impact cycles.

**Dry Samples**	**Peak Load (N)**	**Displacement at Maximum Load (mm)**	**Stiffness (N/mm)**
Non-impacted (raw)	1140	1.72	840
S2	520	1.12	550
S4	729	1.32	698
S6	472	1.00	522
S8	524	1.47	538
**Wet Samples**	**Peak Load (N)**	**Displacement at Maximum Load (mm)**	**Stiffness (N/mm)**
S0	254	0.90	380
S1	360	1.42	270
S3	411	1.96	242
S5	450	2.92	183
S7	470	1.22	390

## Data Availability

Not applicable.
